# NiCo-MOF Nanospheres Created by the Ultra-Fast Microwave Method for Use in High-Performance Supercapacitors

**DOI:** 10.3390/molecules28145613

**Published:** 2023-07-24

**Authors:** Xing Yang, Xin Zhang, Ning Yang, Lei Yang, Wanglong Wang, Xing Fang, Qing He

**Affiliations:** 1 Key Laboratory of Air-Driven Equipment Technology of Zhejiang Province, Quzhou University, Quzhou 324000, China; yang7092481@163.com (X.Y.); 18966410637@163.com (L.Y.); 2 Department of Mechanical Engineering, Zhejiang University of Technology, Hangzhou 310058, China; 2112102228@zjut.edu.cn (X.Z.); 221122020226@zjut.edu.cn (W.W.); 3 Department of Materials Science and Engineering, Tianjin University of Technology, Tianjin 300384, China; 20200988yangning@stud.tjut.edu.cn

**Keywords:** microwave, NiCo-MOF, supercapacitor, asymmetric supercapacitors

## Abstract

Metal-organic frameworks—through the use of creative synthetic designs—could produce MOF materials with excellent porosity, stability, particle microstructures, and conductivity, and their inherent characteristics—including their porosity and controllable structure—may result in an immense number of prospects for energy storage. In this paper, a nanosphere-like NiCo-MOF was effectively manufactured via an ultra-fast microwave technique. Additionally, the ideal synthesis conditions of the NiCo-MOF were investigated by adjusting the microwave output power and microwave reaction time. Under the reaction conditions of a 600 W microwave and a 210 s microwave reaction time, the NiCo-MOF exhibited an excellent capacitance of 1348 F/g at a current density of 1 A/g and an 86.1% capacity retention rate at 10 A/g. In addition, self-assembled NiCo-MOF/AC asymmetric capacitors showed a splendid energy density of 46.6 Wh/kg and a power density of 8000 W/kg.

## 1. Introduction

Given the rising need for renewable energy, the progression of clean and environmentally friendly energy has evolved into the center of current research in light of the current energy predicament. An innovative technique for the energy storage technology known as supercapacitors (SCs) features an exceptional power density and cycle stability, can efficiently convert and store energy, and has been applied to electric and hybrid electric vehicles [1,2,3]. The low energy density of supercapacitors nevertheless, to some extent, restricts their commercial viability. In accordance with the energy storage theory of supercapacitors [4,5], by expanding the voltage window of supercapacitors and the capacitance of electrode materials, the energy density of supercapacitors could get further elevated [6,7]. As the core component of supercapacitors, the performance of supercapacitors as a whole has been confirmed to be considerably affected by their electrode material [8]. Under the current situation, innovative materials for electrodes that have greater energy and power densities pressingly require invention to boost the practical use of supercapacitors [9,10].

Currently, carbon materials, conductive polymers, and transition metal compounds (such as nitrides, sulfides, phosphides, and oxides) are the primary study targets for electrode materials. The outstanding electrical conductivity of carbon materials and conductive polymers has garnered a lot of intrigue, as has their good chemical stability and low price—but their low specific capacitance limits their development. Transition metal compounds are considered promising electrode materials, with a variety of states and high theoretical specific capacitance. However, their lengthy production process and high costs also limit their practical applications [11,12,13,14,15].

Known as metal–organic frameworks (MOFs), transition metal ions and organic ligand junctions make up this type of porous material. Their structural adjustability allows for the flexible design of chemical structures, resulting in excellent MOF materials with a large specific surface area, high porosity, and adjustable structure [16,17]. In particular, a large number of organic functional groups from MOF materials could efficiently improve ion transport and electrochemical performance through a donor-acceptor synergic effect, holding one heteroatom with another and delocalizing the lone pairs of electrons in MOF materials. In addition, abundant heteroatoms (O) can be found in MOF electrode materials, which can be employed as the active site for electrolyte ions in the electrodes [18]. Therefore, MOF materials containing organic functional groups are regarded as the driving force for superior performance. Due to their considerable specific capacitance and adaptable structural features, these materials present enormous possibilities for energy storage [19,20]. For example, Qu et al. [21] used the solvothermal approach to create a novel type of pillared Ni-MOF; the results showed that pillared Ni-MOFs possess a value capacitance with 552 F/g at 1 A/g. By altering the experimental temperatures, Xuan et al. [22] produced a Co-MOF utilizing the solvothermal route; it presented an impressive capacity of 952.5 F/g at 0.25 A/g.

Through recent research, experts have found that, compared with monometallic MOFs, binary MOFs could significantly enhance electrochemical behavior by virtue of the synergistic relationship between bimetals and additional redox processes. Specifically, in bimetal NiCo-MOFs, while the element Ni can encourage the electrochemical activity of the electrode, the Co element can simultaneously lower the charge transfer resistance and strengthen the stability of the compound—thereby improving the specific capacity and rate performance [23,24,25,26]. Investigators such as Sun et al. [27] have used a solvent-controlled technique to successfully create bimetal MOFs that are amino-functionalized with various morphological characteristics. Compared with Ni-MOFs, NiCo-MOFs show a better specific capacitance of 1126.7 F/g at 0.5 A/g. More particularly, the higher capacitance of NiCo-MOF nanosheets was investigated by Wang et al. [28], who constructed extremely thin NiCo-MOF nanosheets at natural temperatures by adopting a simple ultrasonication process. The unique nanosheet-like structure manifested exceptional electrochemical properties, with an excellent capacitance of 1202.1 F/g at 1 A/g. Furthermore, further research has certified that the performance of MOF materials is significantly dependent on their microtopography. Based on the previously reported literature, Du et al. [29] proposed a rapid hydrothermal approach to produce NiCo-MOF particles that resemble flowers and have a size between 5 and 12 µm, which achieved a great capacitance of 927.1 F/g at 1 A/g. A sort of NiCo-MOF with a hollow structure resembling a dandelion was created by Gao et al. [30] through a simple hydrothermal approach. The NiCo-MOF possessed a dandelion-like hollow structure in the range of diameters from 3 to 10 µm, and it exhibited an attractive specific capacitance of 758 F/g at 1 A/g. These studies proved that the performance of MOF materials is significantly dependent on their microtopography, and that the specific morphologies or shrinking of the size of MOFs can maximize the quantity of active sites that are visible on the surface of MOF materials for redox reactions; furthermore, this may simultaneously provide a shorter pathway for electrolyte ion diffusion and charge transfer. Thus, MOF electrode materials with specific morphologies and particles of smaller sizes are expected to show a better capacitive performance [31,32].

In recent years, more and more professionals in numerous fields have grown intrigued with the microwave approach because of its extremely fast reaction efficiency, low experimental costs, and excellent product performance. It is worth noting that the microwave method is an effective way of synthesizing materials with smaller particle sizes and high purities [33,34,35,36,37,38]. Hence, in this work, we successfully prepared NiCo-MOF nanospheres by an ultra-fast microwave method, and further studied the electrochemical performance of different synthesis routes via alteration of the microwave power and microwave reaction time. The results indicated that NiCo-MOF nanospheres synthesized at 600 W and 210 s exhibited an optimal specific capacitance of 1348 F/g at 1 A/g and 60% capacitance retention after 2000 cycles. In addition, the assembled asymmetric supercapacitor devices revealed an impressive energy density of 46.6 W h/kg and power density of 8000 W/kg.

## 2. Results and Discussion

### 2.1. Characterization

**Figure 1:**
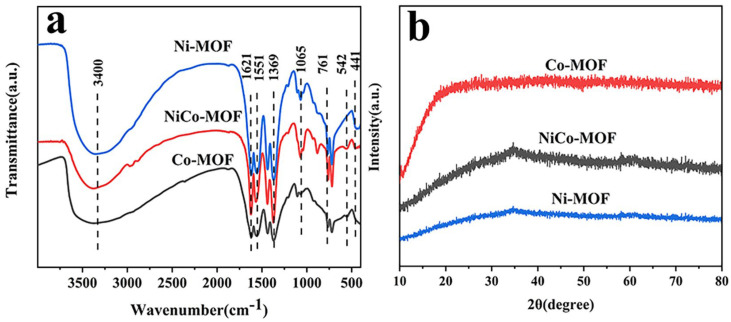
(**a**) FT−IR spectra, (**b**) XRD patterns for Ni-MOF, NiCo-MOF, and Co-MOF.

The infrared spectrum for the Ni-MOF, CO-MOF, and NiCo-MOF are displayed in Figure 1a. A stretching vibration of an -OH functional group was represented by a broad peak at 3400 cm^−1^. The peaks at 1369 cm^−1^ and 1621 cm^−1^ matched to the vs. (-COO) and vas (-COO) of carboxyl from H_3_BTC, certifying that -COO groups were able to effectively coordinate with the metal center in bidentate mode. A series of characteristic peaks at 1065 cm^−1^, 761 cm^−1^, and 1551 cm^−1^ were related to the stretching vibrations of the aromatic ring. For the low wave-number region, the positions located at 441 cm^−1^ and 542 cm^−1^ were derived from stretching vibrations caused by Ni–O and Co–O bonds. This result is strong evidence for the formation of NiCo-MOF [39,40]. Furthermore, the XRD pattern in Figure 1b makes it perfectly obvious that there was almost no diffraction peak in the Ni-MOF, NiCo-MOF, or Co-MOF—indicating their amorphous nature. Based on previously reported work, amorphous MOFs have displayed excellent electrochemical performance; it has been confirmed that amorphous materials are advantageous for the deeper diffusion of their electrolyte ions—therefore, this can effectively improve the electrochemical properties of MOF electrodes [41,42].

**Figure 2:**
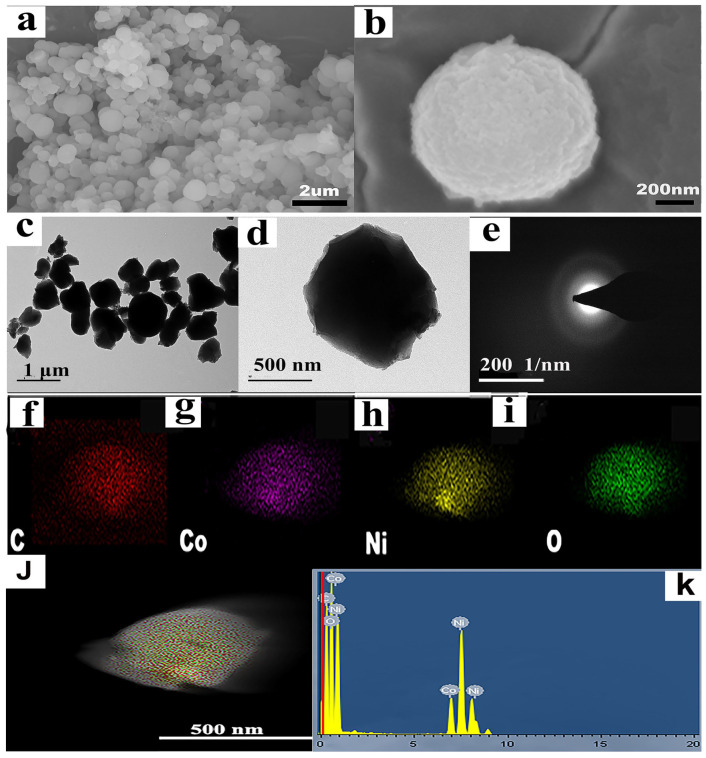
(**a**,**b**) SEM images of NiCo-MOF; (**c**,**d**) TEM images of NiCo-MOF; (**e**) SAED of NiCo-MOF; (**f**–**j**) C, CO, Ni, and O element mapping of NiCo-MOF; (**k**) EDS of NiCo-MOF.

The SEM photograph in Figure 2a,b depicts the morphology of NiCo-MOF, which displayed uniform NiCo-MOF nanospheres of approximately 500 nm in diameter on average. The microstructure of the NiCo-MOF sample was further analyzed via TEM, and the corresponding pictures are displayed in Figure 2c,d. What can be seen is that NiCo-MOF was formed by the nanospheres of particle sizes that ranged between 300 and 800 nm. Specifically, the nanospheres were solid and uniformly distributed. The SAED diagram is shown in Figure 2e; the electron diffraction diagram presented a wide and diffused halo ring—indicating the amorphous nature of the NiCo-MOF, which also corresponded with the XRD results. The concentration of elements and EDS spectrum of the NiCo-MOF sample are presented in Figure 2f–k. The results indicated that NiCo-MOF is free of additional elements and only comprises the components Ni, Co, C, and O; these elements were distributed evenly throughout the NiCo-MOF, further supporting the viability of NiCo-MOF synthesis via the microwave method.

**Figure 3:**
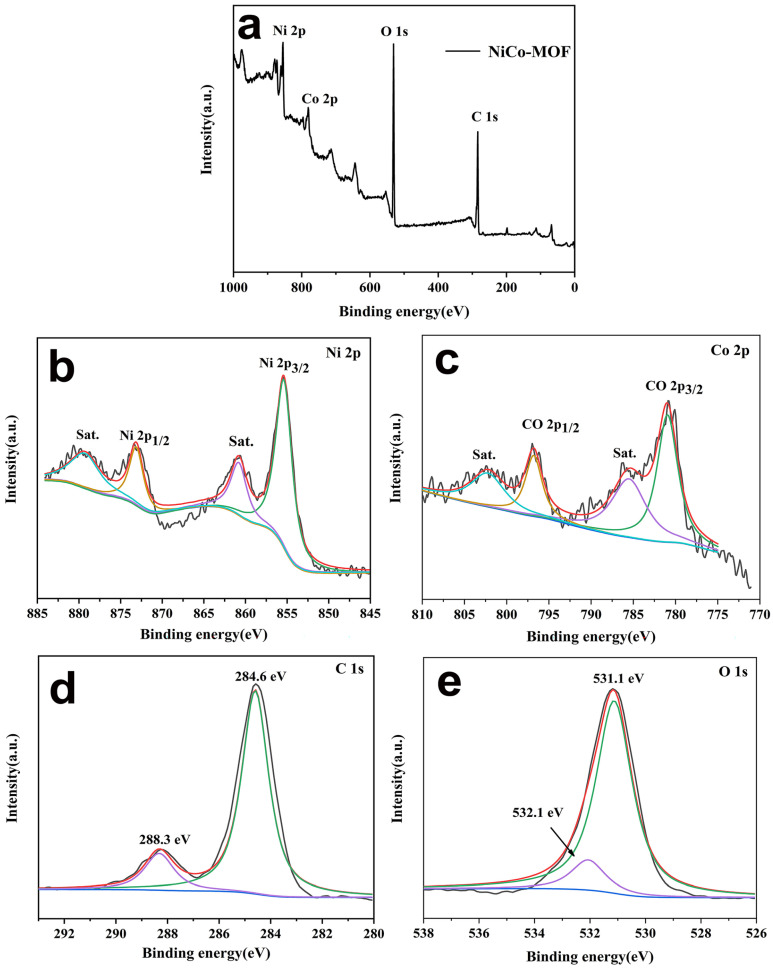
XPS survey spectrum for NiCo-MOF, (**a**) survey scan spectrum, (**b**) Ni 2p, (**c**) Co 2p, (**d**) C 1 s, and (**e**) O 1 s.

The valence states of the elements in the NiCo-MOF material were analyzed via the XPS test, and results appear in Figure 3. The survey spectrum for the NiCo-MOF sample is presented in Figure 3a. The results indicate that the elements C, O, Co, and Ni were detected on the surface of the NiCo-MOF—Table 1 states each element content level (At %). This demonstrates that Ni/Co has an atomic ratio of roughly 1.89. The elements C 1 s, O 1 s, Ni 2p, and Co 2p were further fitted and analyzed. As shown in Figure 3b, the Ni 2p spectrum showed its principal peaks at 855.2 eV and 872.8 eV with a spin-energy separation of 17.6 eV, related to the spin orbits of Ni 2p_3/2_ and Ni 2p_1/2_, respectively. Additionally, two broad peaks at 861 eV and 879.1 eV were attributed to shake-up satellites (Sat.) of Ni 2p_3/2_ and Ni 2p_1/2_, indicating the characteristic bands of Ni^2+^. Similarly, Figure 3c shows the Co 2p spectrum; the strong peaks located at around 781 eV and 796.8 eV with a spin-energy separation of 15.8 eV can be assigned to Co 2p_3/2_ and Co 2p_1/2_, and were accompanied by a group of broad peaks centered at 802.7 eV and 785.4 eV that corresponded to shake-up satellites characteristic of Co^2+^. These above analyses fully proved that the valence states of Ni^2+^ and Co^2+^ were present in the NiCo-MOF nanospheres. In Figure 3d, two main peaks of the C 1 s spectrum are seen at 284.6 eV and 288.3 V—corresponding to the binding energy that exists in C–C=C and O–C=O bonds, respectively. In Figure 3e, the spectrum with the binding energy for O 1 s was connected with M–OH and -OH bonds at 531.1 eV and 533.3 eV [28,30,31].

### 2.2. Electrochemical Properties

**Figure 4:**
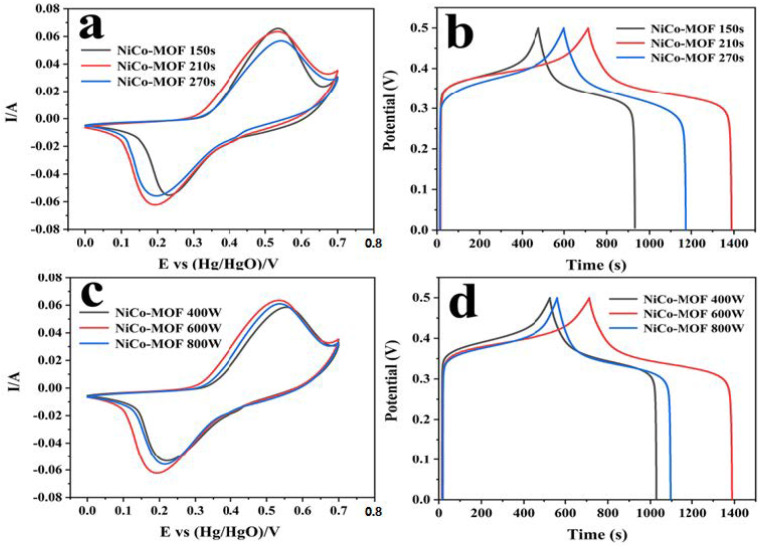
Electrochemical performance of NiCo-MOF under different reaction conditions: (**a**,**c**) CV curves at a scan rate of 30 mV/s; (**b**,**d**) GCD curves at a current density of 1 A/g.

The electrochemical performance of the NiCo-MOF was further explored by adjusting the microwave time and power, and is shown in Table 2. Through the conventional three-electrode exam, the electrochemical performance of the produced electrodes was evaluated through a 2 M KOH electrolyte, and the corresponding picture can be viewed in Figure 4. The synthesized NiCo-MOF at 600 W and 210 s presented the maximum CV sealing area and discharge time. When the reaction duration was 270 s or the microwave reaction power reached 800 W, the specific capacitance reduction may have been attributable to the higher energy in the reaction system causing the faster formation of nanoparticles—resulting in the poor uniformity and agglomeration of nanoparticles. Inversely, a lower microwave power and microwave time may be detrimental to particle integrity due to inadequate reactions occurring; as such, the NiCo-MOF would have fewer active sites for electron transport and thus a lower capacitance [9,43].

**Figure 5:**
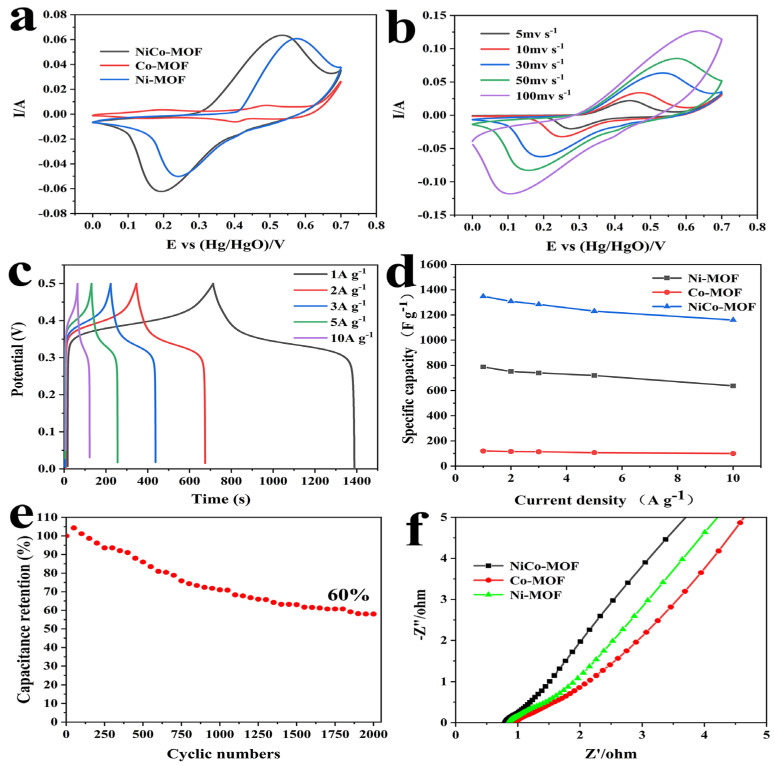
(**a**) Comparison of CV curves of NiCo-MOF, Co-MOF, and Ni-MOF at 30 mV/s; (**b**) CV curves of NiCo-MOF with varied scan rates; (**c**) GCD curves of NiCo-MOF at different current densities; (**d**) specific capacitance of NiCo-MOF, Co-MOF, and Ni-MOF at different current densities; (**e**) cycling stability of NiCo-MOF at 10 A/g, and (**f**) Nyquist plots of NiCo-MOF, Co-MOF, and Ni-MOF.

In order to further evaluate their electrochemical performance, NiCo-MOF, Ni-MOF, and Co-MOF were prepared under identical circumstances and utilized as comparison electrodes. The CV, GCD, and EIS are summarized in Figure 5. In Figure 5a, the CV curves of the Ni-MOF, Co-MOF and NiCo-MOF electrodes are compared by employing a 30 mV/s scan rate. It was evident that all the CV curves showed a pair of strong redox peaks, which were derived from the redox reactions of Co^2+^/Co^3+^ and Ni^2+^/Ni^3+^ in the KOH electrolytes. Apparently, the biggest CV curve area was visible in the NiCo-MOF electrode CV curve, which means that it had a larger charge storage capacity—convincingly proving that the excellent synergism between Ni–Co bimetals could greatly improve electrochemical behavior. Figure 5b displayed all the CV curves between a potential range of 0 and 0.7 V at various scan rates. It was found that the shape of the CV curves remained mostly unaltered as the scanning rate increased, and there was still an obvious redox peak at scan rates of up 100 mV/s—indicating exceptional rate capabilities and reversibility for the NiCo-MOF.

Figure 5c presents the GCD curves of the NiCo-MOF at current densities of 1 to 10 A/g with a potential window of 0 to 0.5 V. At all current densities, the approximately symmetric features of the GCD curves indicated that the NiCo-MOF electrode possessed outstanding reversible electrochemical behavior. Next, according to the test results of the GCD, calculations were carried out to determine each electrode’s specific capacities at various current densities, and the results are illustrated in Figure 5d. The NiCo-MOF electrode had the highest specific capacities, which were 1348 F/g, 1328 F/g, 1284 F/g, 1230 F/g, and 1160 F/g at the current densities of 1–10 A/g, respectively—which was much higher than the Ni-MOF and Co-MOF electrodes. In addition, at a high current density of 10 A g^−1^, the NiCo-MOF, Ni-MOF, and CO-MOF electrodes still maintained desirable capacitances of 1160 F/g, 637 F/g, and 100 F/g respectively—which was about 86.1%, 80.8%, and 83.3% of their capacitance at 1 A/g. Moreover, in comparison to earlier reports of Ni/CO-MOF supercapacitor electrodes in Table 3, these results also proved that the synergistic action of Ni-Co bimetals could considerably boost the rate capacity and charge storage capacity of electrodes.

Furthermore, the impedance of NiCo-MOF, Ni-MOF, and Co-MOF were analyzed via the EIS impedance test; the corresponding Nyquist diagram is shown in Figure 5f. In the high-frequency region, the intercept on the real axis represented the internal resistance (Rs); the Rs value of the NiCo-MOF was 0.80 Ω, which was lower than the Ni-MOF (0.89 Ω) and Co-MOF (0.90 Ω). The semicircle diameter represented the Faraday charge transfer impedance (Rct) at the interface of the electrode and electrolyte; no sample exhibited a significant semicircle, implying a small charge transfer resistance—this was mainly because amorphous structures are able to generate abundant free holes and reduce the charge transfer resistance [41]. The slope of the line in the low-frequency range was directly connected to the diffusion of electrolyte ions. The NiCo-MOF electrode was closer to a vertical slope than the other electrodes, which means that it had a quicker ion diffusion rate. The electrochemical stability of the NiCo-MOF electrodes was then assessed by employing charge–discharge tests at 10 A/g. In accordance with Figure 5e, after 2000 cycles, the capacity retention rates of the NiCo-MOF electrodes remained at 60% of the initial capacitance.

To further estimate the actual applications of NiCo-MOF electrodes for electrochemical energy storage devices, the NiCo-MOF was employed as the positive electrode and activated carbon (AC) was deployed as the negative electrode to fabricate an asymmetric supercapacitor (ASC), and its electrochemical performance in 2 M KOH electrolyte was investigated. The coating mass of the positive and negative electrode should be determined via the following equation in accordance with the positive and negative charge balance principle:

(1) $$\frac{m^{+}}{m^{-}}=\frac{C^{-}\Delta V^{-}}{C^{+}\Delta V^{+}}$$

where *C*+, *C*^−^, Δ*V*+, and Δ*V*^−^ stand for the specific capacitance (F/g) and discharge voltage (Δ*V*) of the positive and negative electrodes, respectively. In accordance with the calculations, the mass ratio of the materials for the positive/negative electrodes was approximately 1:2. The coating mass of the final positive electrode was about 1.2 mg cm^−1^ and the coating mass of the negative activated carbon was about 2.4 mg cm^−1^, which was close to the calculated result.

**Figure 6:**
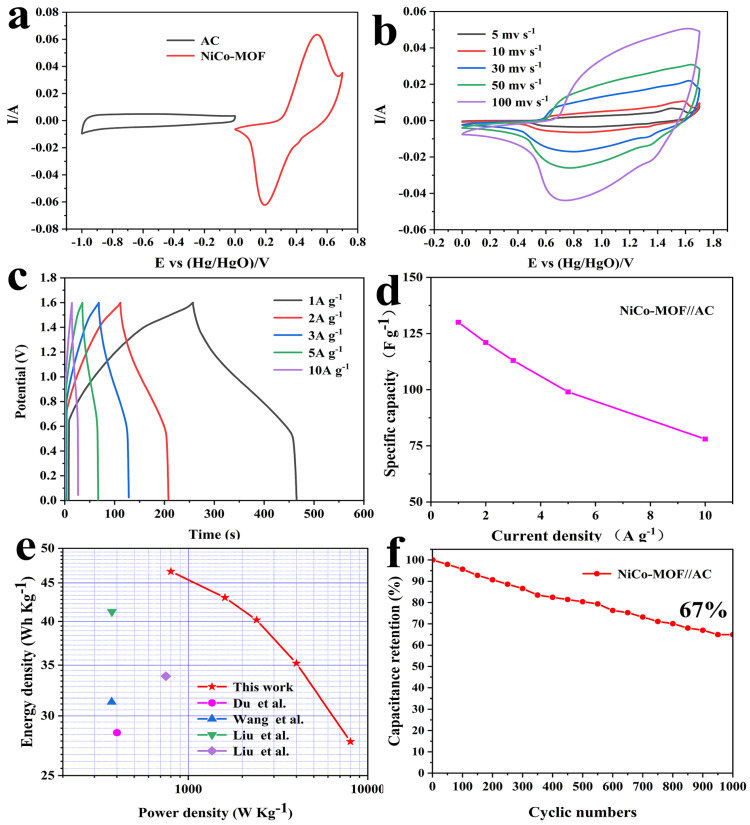
(**a**) CV curves of AC and NiCo-MOF electrodes at a scan rate of 30 mV/s; (**b**) CV curves of the NiCo-MOF//AC at different scan rates; (**c**) GCD curves and (**d**) the specific capacitance of the NiCo-MOF//AC at different current densities; (**e**) Comparison of power and energy densities; (**f**) Cycling stability of the asymmetric supercapacitor. Red star-this work, pink circle [29], blue triangle [45], green triangle [31], purple rhomb [46].

To confirm the usable potential window of the NiCo-MOF//AC device, measurements were performed on the NiCo-MOF and AC with a scanning rate of 30 mv/s. Figure 6a demonstrates that the potential window range for the AC electrode was between −1.0 V and 0 V, whereas the range for the NiCo-MOF electrode was between 0 and 0.7 V; these results indicated that the possible voltage window of the NiCo-MOF//AC device may be 1.7 V. Moreover, the CV curve of the NiCo-MOF had obvious redox peaks, which represent a battery-type electrode material. The shape of the AC was nearly a rectangle, which belongs to the typical electric double layer-type electrode material. Figure 6b exhibits the CV curves for the ASC device with scan rates ranging from 5 to 100 mv/s. The CV curves included double layer capacitances and Faradaic redox behavior and the corresponding potential window could be further enlarged to 1.7 V. With the increase of the scanning rate, the CV curve for the ASC device did not significantly change in shape, indicating its ability to quickly charge and discharge.

The GCD curves for ASC at varying current densities (1–10 A/g) are displayed in Figure 6c. As indicated by the approximately symmetric GCD curves, the GCD curves indicated that asymmetric supercapacitors possess a favorable excellent electrochemical reversibility. Figure 6d displays a calculation of the specific capacitances—remarkably, the ASC device with NiCo-MOF//AC presented an outstanding capacitance of 131 F/g at 1 A g^−1^, and maintained 78 F/g at 10 A/g. Computing the GCD curve, which can be obtained by adopting the formulae that are listed below, the values of the energy density and power density were able to be calculated:

(2) $$E=\frac{C\times {\left(\Delta V\right)}^{2}}{7.2}$$

(3) $$P=\frac{3600\times E}{\Delta t}$$

In which *E* is the energy density (Wh/kg), *C* is the specific capacitance of ASC (F/g), Δ*V* is the discharge potential window (*V*), *P* is the power density (W/kg), and Δt is the discharge time (s); detailed numerical values are shown in Figure 6e. The power density can be maintained at 800 W/kg when the energy density reaches 46.6 Wh/kg; the energy density remains at 27.73 Wh/kg even though the power density reaches up to 8000 W/kg. This result is superior to most recent reports of Ni/CO-MOF electrode materials, indicating that they have an extensive number of practical application possibilities. In addition, for the purpose of evaluating the cyclic stability of the ASC, 1000 cycles were carried out under conditions of 10 A/g. The specific capacitance of the ASC device ultimately stayed at 67% of the original level over 1000 cycles, as illustrated in Figure 6f—suggesting an exceptional cycle stabilization performance.

## 3. Materials and Methods

### 3.1. Materials

Cobalt chloride hexahydrate (CoCl_2_·6H_2_O), nickel chloride hexahydrate (NiCl_2_·6H_2_O), trimesic acid, 1,3,5-Benzenetricarboxylic acid (H_3_BTC), and N-N-Dimethyformamide (DMF) were obtained from MACKLIN chemical reagent Co., Ltd. (Shanghai, China). The sources of the ethylene glycol and potassium hydroxide were Taicang Hu Test reagent Co., Ltd. (Suzhou, China). and the acetylene black and polyvinylidene fluoride were supplied by Tian Jin Chemical Technology Co., Ltd. (Tianjin, China). No additional processing was done to any of these products before usage.

### 3.2. Preparation for NiCo-MOF Electrodes

**Figure 7:**
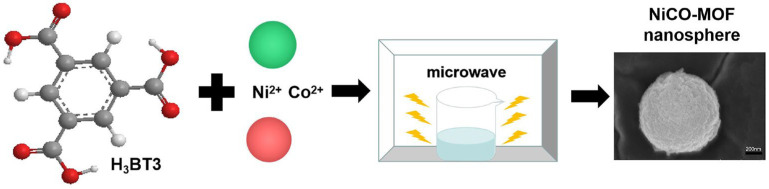
Synthesis of NiCo-MOF.

As shown in Figure 7, NiCo-MOF were prepared by the ultra-fast microwave method: 1.14 g CoCl_2_·6H_2_O, 1.14 g NiCl_2_·6H_2_O, and 0.7 g H_3_BTC were dissolved into 200 mL of mixed solution, in which the volume ratio of DMF to ethylene glycol was 1:1. The mixture was then stirred for an additional hour until it formed a homogenous solution. After this, the combination was placed in a microwave oven (PANASONIC NN-GF352 M, 2450 MHz, Shanghai, China), where it was heated for 210 s at a power of 600 W. The suspension was filtered, then alternately cleaned with DMF and deionized water until the filtrate was colorless for a long time. Then, the collected product was transferred to a vacuum oven, drying for 12 h at 80 °C and named NiCo-MOF. Aside from this, a series of NiCo-MOFs with different variables were created under similar procedures.

The following procedure was employed to prepare the working electrodes: nickel foam (1 × 1 cm^2^) was used as the collector fluid of working electrode, the acetylene black as a conductive agent, and the PVDF particles were dissolved in NMP as a PVDF adhesive solution. First, the synthesized electrode material (NiCo-MOF) was combined with PVDF and acetylene black at a 1:1:8 mass ratio, and the resulting slurry was then evenly coated onto the nickel foam and allowed to dry for 24 h at 80 °C. About 0.8 to 1.0 mg/cm^2^ of the active substance was coated on the nickel foams, and finally, the electrodes were pressed by a tablet press.

### 3.3. Characterization

The Fourier transform infrared spectrum (FT-IR) method, using a THERMO FISHER NICOLET 6700 from USA, was used to measure the functional groups of the NiCo-MOF. The crystal structure of the NiCO-MOF was analyzed using X-Ray Diffraction (XRD) with a Rigaku ULTIMAIV from Japan. The morphologies of the NiCo-MOFs prepared under different conditions were collected by a scanning electron microscope (SEM, Hitachi-SU-8100, Tokyo, Japan). The composition and valence state of the samples were measured by X-ray photoelectron spectroscopy (XPS, ESCALAB 250XI, Waltham, MA, USA). The transmission electron microscopy (TEM) and energy dispersive spectrum (EDS) were analyzed using a TECNAI G2 F30 from USA.

The electrochemical performance of the NiCo-MOFs was examined by applying a CHI660E electrochemical workstation in 2 M KOH electrolyte. In a three-electrode system, the NiCo-MOF electrodes were employed as the working electrodes; platinum electrodes and Hg/HgO electrodes were used as counter electrodes and reference electrodes, respectively. The cyclic voltammetry (CV) curves scanned across 0 to 0.7 V, and electrochemical impedance spectral (EIS) measurements were conducted in the frequency range of 100 khz–1 hz; the galvanostatic charge–discharge (GCD) was recorded from 1 to 10 A g^−1^, and their specific values were calculated according to the following equation:

(4) $$C=\frac{I\Delta t}{m\Delta V}$$

where *C* represents the specific capacitance (F/g), m represents the mass of the active material (mg), Δ*t* represents the discharge time (s), Δ*V* represents the potential window (*V*), and *I* represents the charge/discharge current (A).

## 4. Conclusions

In summary, nano-spherical NiCo-MOF was successfully prepared using the ultra-fast microwave method. The optimum electrochemical performance of the NiCo-MOF was achieved by adjusting the microwave power and reaction time. With the microwave power set at 600 W and the microwave time at 210 s, the NiCo-MOF electrode showed the highest specific capacity (1348 F/g at 1 A/g), as well as an excellent rate capability (86.1% capacity retention rate at 10 A g^−1^) and cyclic stability (60% capacity retention rate over 2000 cycles at 10 A/g). Besides this, the electrochemical performance of the asymmetric supercapacitor devices were investigated in detail; the asymmetric supercapacitor based on NiCo-MOF as the positive electrode and activated carbon as the negative electrode exhibited an excellent energy density of 46.6 Wh/kg and power density of 8000 W/kg. These results can further expand the applications for amorphous NiCo-MOF materials in supercapacitors.

## Figures and Tables

**Table 1:** Element content obtained from XPS of the sample.

Sample	Element Content (At %)			
	C	O	Ni	Co
NiCo-MOF	56.23%	32.83%	7.16%	3.78%

**Table 2:** NiCo-MOF list with different reaction parameters.

Sample	Microwave Power (W)	Microwave Time (s)
NiCo-MOF	600	210
NiCo-MOF 150 s	600	150
NiCo-MOF 270 s	600	270
NiCo-MOF 400 w	400	210
NiCo-MOF 800 w	800	210

**Table 3:** Capacitance of Ni/Co-MOF materials.

Sample	Electrolyte	Specific Capacitance	Ref
Flower-like NiCo MOF	3 M KOH	927 F/g at 1 A/g	Ref [29]
Dandelion-like NiCo MOF	2 M KOH	758 F/g at 1 A/g	Ref [30]
Ultrathin nanosheets NiCo MOF	2 M KOH	1202.2 F/g at 1 A/g	Ref [28]
Hydrangea-like NiCo MOF	2 M KOH	1056.6 F/g at 0.5 A/g	Ref [40]
Pillar Ni MOF	2 M KOH	552 F/g at 1 A/g	Ref [21]
Urchin-like Co-MOF	3 M KOH	952.5 F/g at 0.25 A/g	Ref [22]
Spherical NiCo-MOF	6 M KOH	715 F/g at 1 A/g	Ref [44]
Nanosphere-like NiCo-MOF	2 M KOH	1348 F/g at 1 A/g	This work

## Data Availability

The data that support the findings of this work are available from the corresponding author upon reasonable request.

## Sample Availability

Samples of the compounds are available from the authors.
